# Supplementary material for: Engineering flexible superblack materials
Source: Nat Commun. 2025 May 19;16:4650. doi: 10.1038/s41467-025-59876-y (PMC12089317; doi:10.1038/s41467-025-59876-y)
Supplement: Supplementary file 1 — Supplementary Information [file 41467_2025_59876_MOESM1_ESM.pdf]

Supplementary Information: Engineering Flexible Superblack Materials

*Yucheng Yang[1], Botond Sánta[1], Ashok Ponnuchamy[2], Edward C. Kinzel[1], Anthony J. Hoffman[2], Matthew R. Rosenberger[1]**

[1]Department of Aerospace and Mechanical Engineering, University of Notre Dame, Notre Dame, IN 46556, United States

[2]Department of Electrical Engineering, University of Notre Dame, Notre Dame, IN 46556, United States

* Corresponding author, mrosenb2@nd.edu

Controlling silicon pillar design through photolithography pattern design

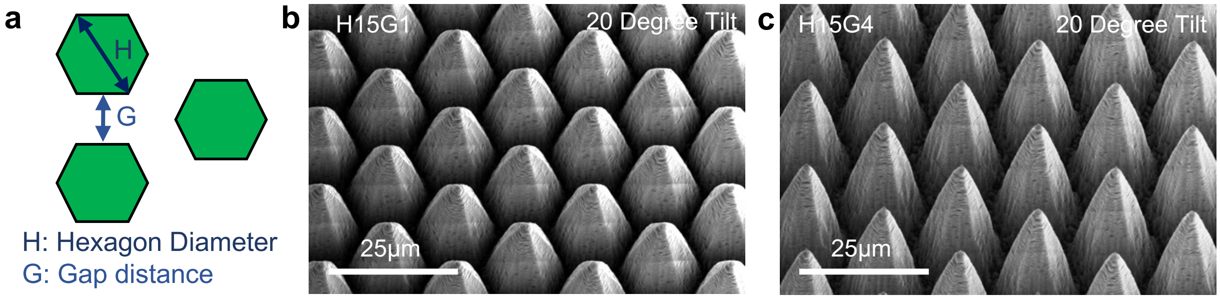

Supplementary Fig.1: Controlling silicon design: (a) photolithography pattern design. Silicon pillar tilted SEM for (b) H15G1 and (c) H15G4.

SEM images of the mold surface after more than 5 castings:

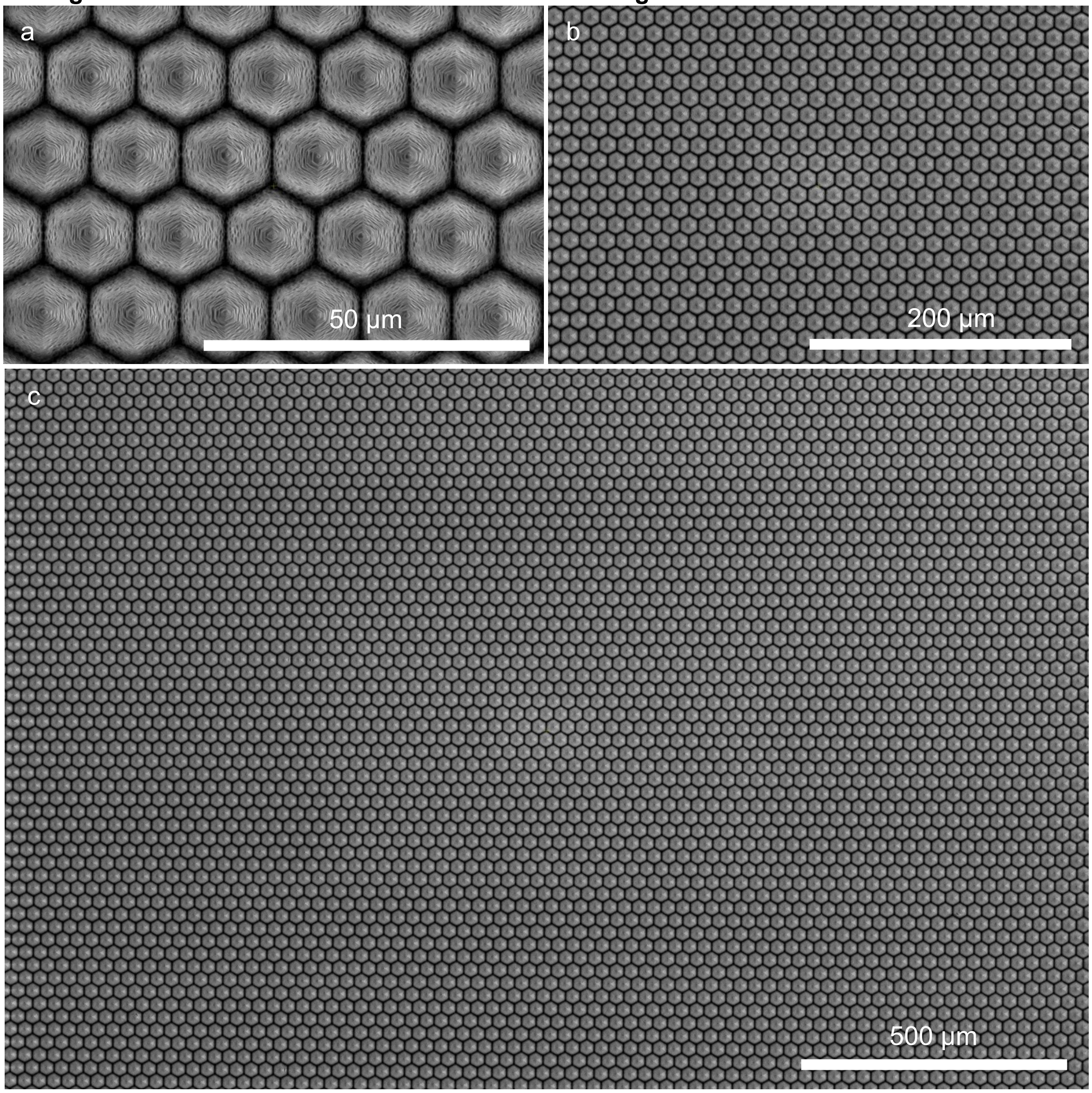

Supplementary Fig. 2: H15G1 SEM images of the mold surface after more than 5 castings at different magnifications (a) 2500X, (b) 500X, and (c) 200X.

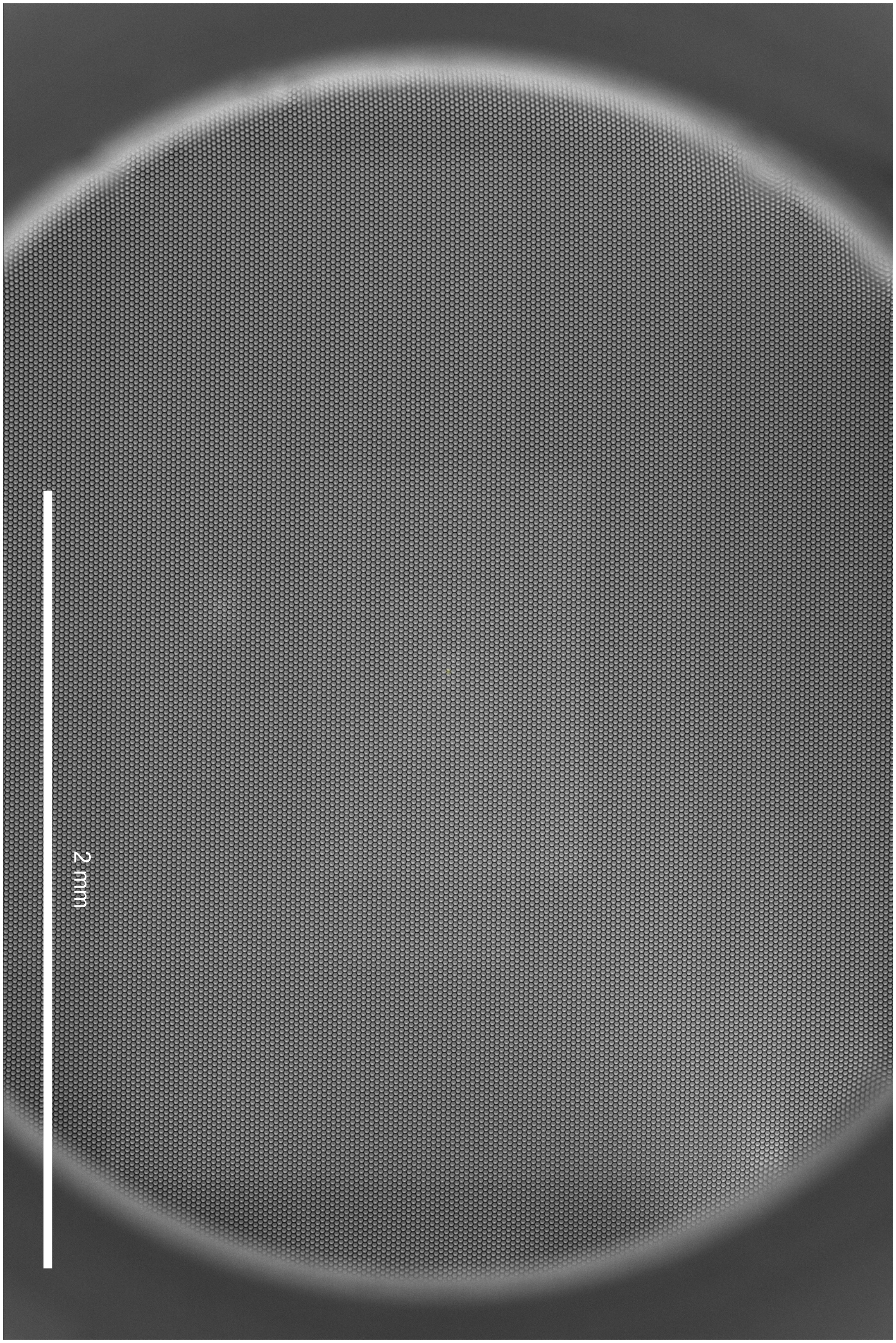

Supplementary Fig. 3: H15G1 SEM images of the mold surface after more than 5 castings at 61X magnifications.

Photography of varying reflectance sample under various shutter speed:
At a shutter speed of 1/50 s, the distinction between H15G2 and H15G3 is evident. H15G2 exhibits an average reflectance of 0.25% across the visible spectrum (400nm to 700nm), whereas H15G3 shows 0.35%. This indicates that under an illumination intensity of ~20,000 lux, differences in reflectance as small as 0.1% can be discerned.

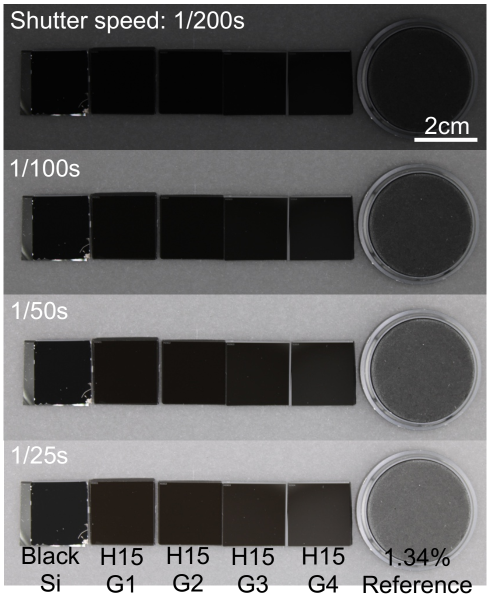

Supplementary Fig. 4: Visually comparing material with different reflectivity at different shutter speed under 21,000 lux illumination. The shutter speed for the top panel to bottom panel varied as follows: 1/200s, 1/100s, 1/50s, and 1/25s. The reflectance spectrum is shown in Fig. 4a.

Quantitative comparison of PDMS reflectance for several castings:
We cast five different PDMS samples from the same R-H15G1 mold and measured the total reflectance at near-normal incidence to quantify repeatability.

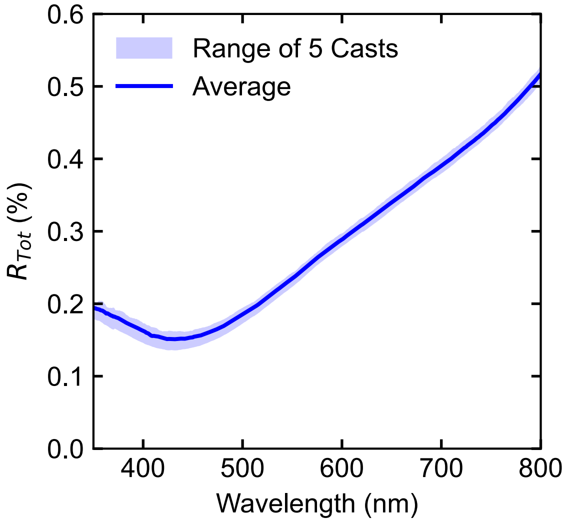

Supplementary Fig. 5: Quantitative comparison of total reflectance at near-normal incidence angle for 5 casts of the same R-H15G1 mold.

Microcavity uniformity:

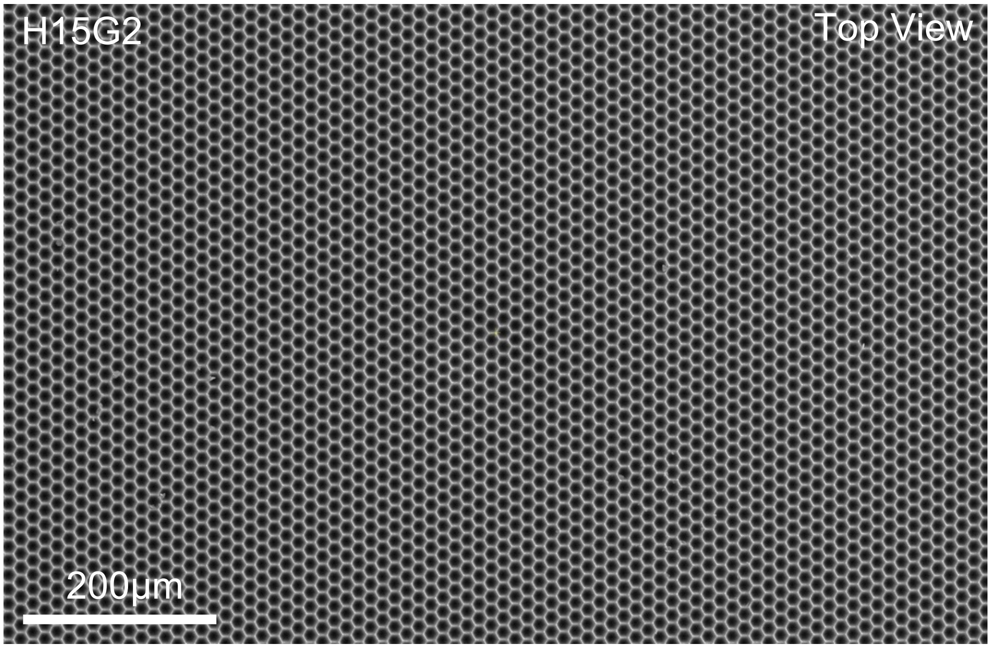

Supplementary Fig. 6: Demonstrating microcavity uniformity across large scale using SEM.

Reflectance characterization techniques:

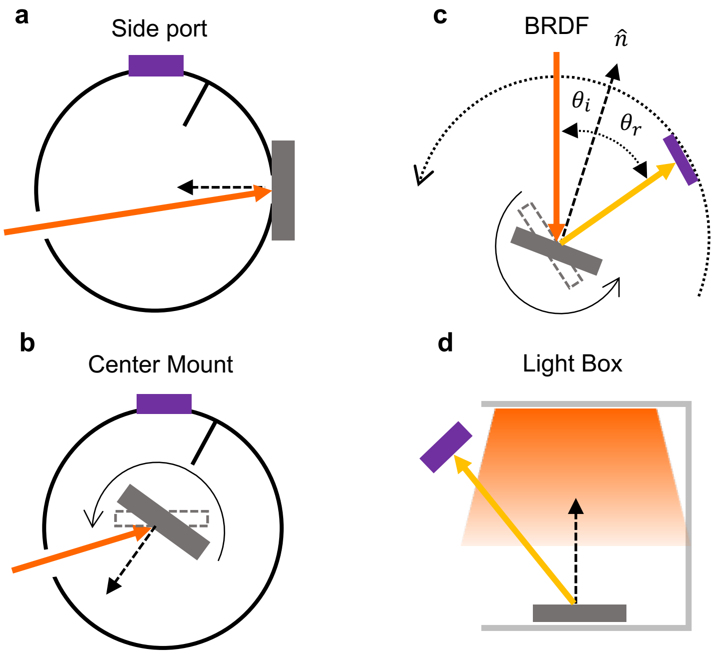

Supplementary Fig. 7. Diagrams for four optical characterization techniques: (a) side port total hemispherical reflectance integrating sphere measurement; (b) center mount angle dependent total hemispherical reflectance integrating sphere measurement; (c) bidirectional reflectance distribution function measurement; (d) Light box photography.

Black Silicon:

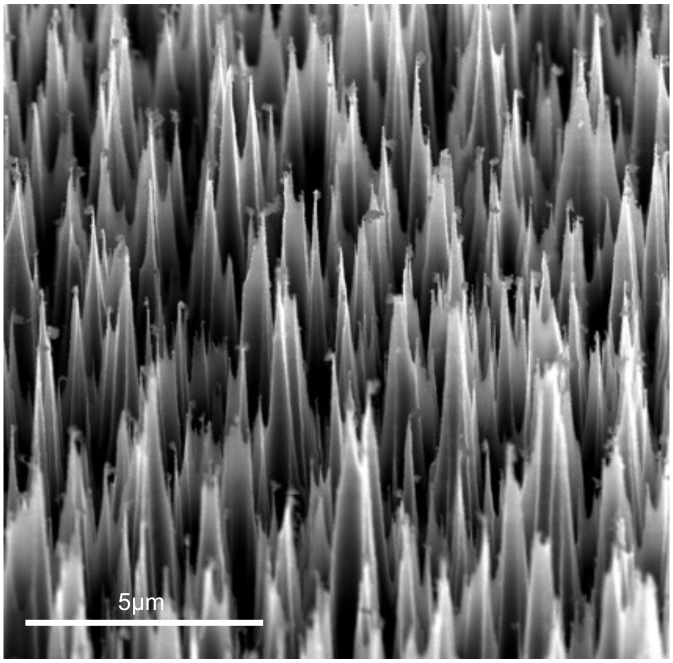

Supplementary Fig. 8. Tilted view SEM image of the black silicon surface.

SEM and CSI measurement on H15 with varying G:
With increasing G, the observed microcavity top surface area increases which is consistent with SEM image (Supplementary Fig. 9a). CSI can show the locations on surfaces that have significant reflectivity at the microscale. We performed CSI measurement on samples with fixed hexagon diameter and varying gap distance (Supplementary Fig. 9b). White regions are the regions without any signal which is likely due to lack of reflected light. This result indicates the manufactured microcavity effectively attenuates light that enters the cavity body.

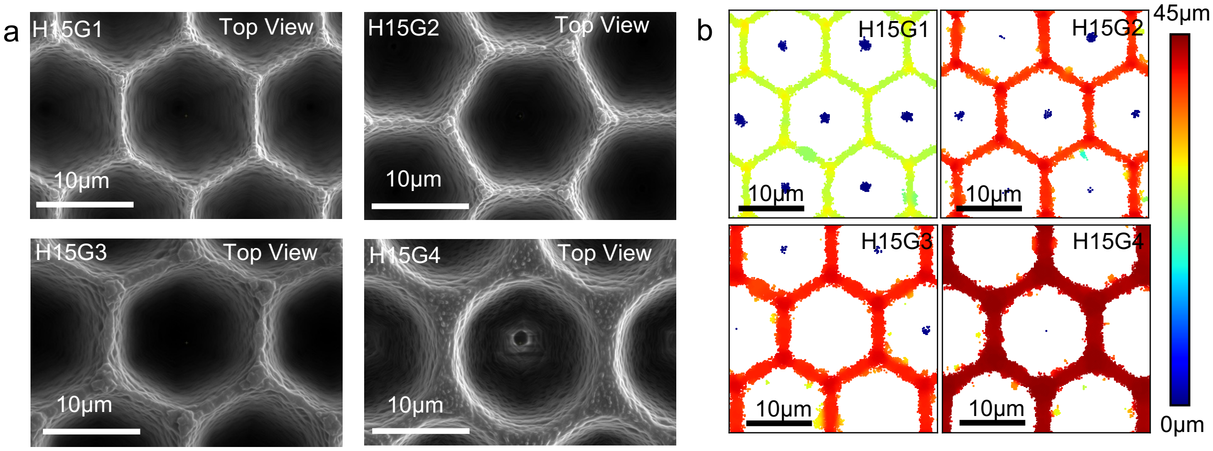

Supplementary Fig. 9. Topography comparison between microcavities with varying gap-distance using (a) SEM and (b) CSI.

Aspect ratio calculation of microcavity with constant G1 and varying H

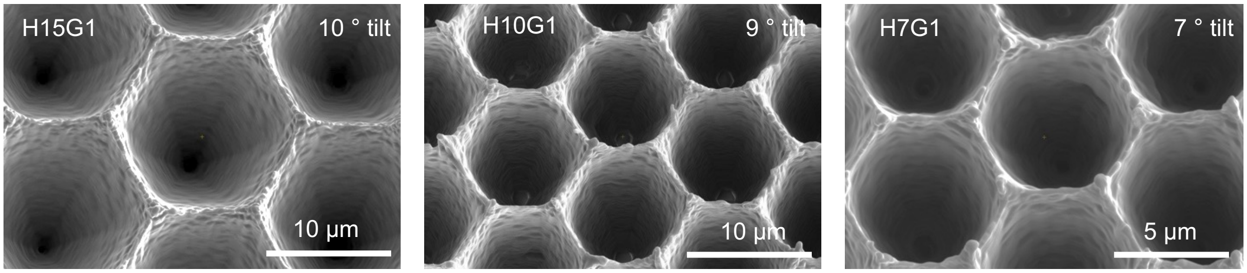

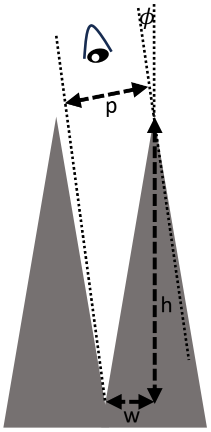

$$h = \frac{P - w * \cos(\phi)}{\sin(\phi)}$$

Supplementary Fig. 10. SEM image and aspect ratio (h/w) calculation method for varying hexagon while keeping the same gap.

Supplementary Table 1. Aspect ratio measurement data and calculation based on Supplementary Fig. 10.

Sample Name	w (μm)	p (μm)	ϕ (°)	h (μm)	h/w
H15G1	6.5	10.5	10	24	3.6
H10G1	4.3	8.3	9	26	6
H7G1	3.0	5.5	7	20	6.7

Total reflectance and BRDF at near normal incident on samples with constant G1 and varying H

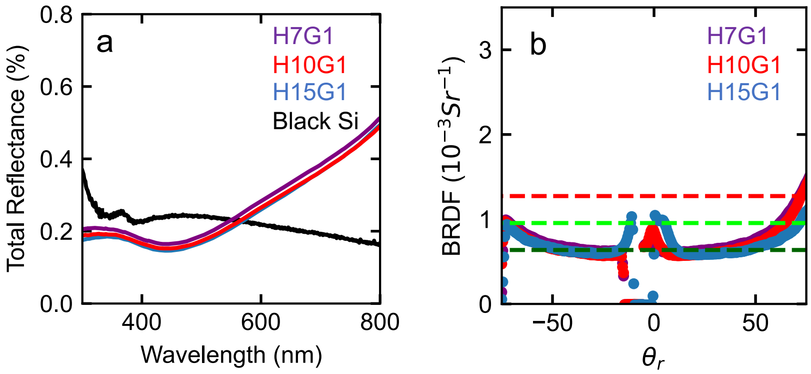

Supplementary Fig. 11. Optical characterization of microcavity samples with constant G and varying H using (a) integrating sphere (b) 2D-BRDF.

Dual beam 60mm integrating sphere precision and accuracy:

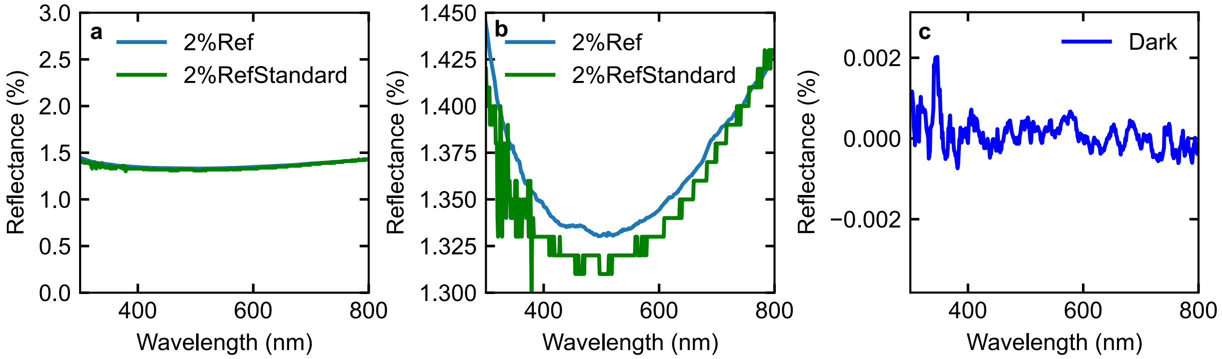

Supplementary Fig. 12. 60 mm side port integrating sphere reflectance measurement accuracy and precision validation: (a) comparison between our measurement (blue) to third party measurement (green). (b) zoomed-in version of (a). (c) dark measurement.

150mm side port integrating sphere precision:

Supplementary Table 2. 150 mm integrating sphere side port measurement: The average detector current and standard deviation of the detector current of 60 measurements with 1 second integration time. The calculated dark reflectance is ~100 times lower than the reflectance of H15G1.

	**150 mm Integrating Sphere Side Port Measurement (60 measurements with 1 second integration time)**				
	99% Standard (detector current)		Dark (detector current)		
Wavelength (nm)	Average (μA)	Standard Deviation (μA)	Average (μA)	Standard Deviation (μA)	Dark Reflectance (%)
450	6.208	0.012	0.000069	0.000005	0.001104
500	7.514	0.006	0.000077	0.000029	0.001016
532	16.193	0.022	0.000158	0.000007	0.000967
550	14.306	0.024	0.000169	0.000014	0.001172
600	18.107	0.007	0.000311	0.000005	0.001703
650	19.553	0.017	0.000373	0.000007	0.001890
700	23.123	0.015	0.000873	0.000013	0.003737
750	23.570	0.010	0.000708	0.000012	0.002974

Additional BRDF measurements:

The BRDF can be calculated from the following equation: when the angle of incidence θ_i is fixed:

$$BRDF(\theta_r) = \frac{P_{sig}}{P_{ref}\cos(\theta_r)\delta\Omega_r}$$

Where θ_r is the polar angle measured from the normal vector of the sample plane corresponding to the location of the power detector (Fig, 4d). P_{sig} is the measured power signal. P_{ref} is the reference power which is the power of the laser. $\delta\Omega_r$ is the solid angle subtended by the power detector. For a fixed laser wavelength, fixed θ_i, and assuming Azimuthal symmetry around the normal vector along the specular angle, the BRDF is only a function of θ_r. BRDF has units of inverse steradian (Sr^{-1}). To compare with the integrating sphere measurement, we plot BRDF*Cos(θ_r), shown in Supplementary Fig. 13 a,b. The total reflectance calculated from the BRDF is compared against side port integrating sphere measurement shown in Supplementary Fig. S13 c,d.

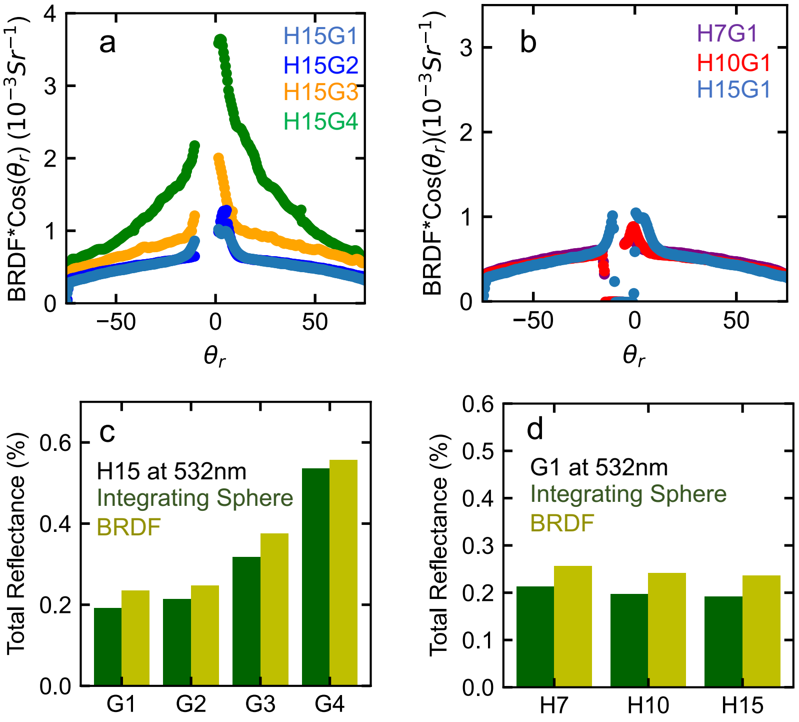

Supplementary Fig. 13. Demonstration of reflectance characterization consistency between measurements: Total reflectance comparison between BRDF calculated and integrating sphere measurement: BRDF*Cos(θ_r) for sample (a) constant H and varying G (b) constant G and varying H. The total reflectance comparison between the integrating sphere measurement and calculated using BRDF measurement at 532nm laser illumination for samples with (c) constant H and varying G and (d) constant G and varying H.

150mm center mount integrating sphere precision:

Supplementary Table 3. 150 mm integrating sphere center mount measurement average detector current and standard deviation of the detector current of 60 measurements with 1 second integration time. The reduction in detector current for 99% standard and increase in the detector current for dark measurement is due to the presents of highly absorbing material and clip mount sample holder present in the integrating sphere.

	**150 mm Center Mount Integrating Sphere Measurement (60 measurements with 1 second integration time)**				
	99% Standard (detector current)		Dark (detector current)		
Wavelength (nm)	Average (µA)	Standard Deviation (µA)	Average (µA)	Standard Deviation (µA)	Dark Reflectance (%)
450	2.931	0.011	0.000891	0.000004	0.030
550	6.665	0.017	0.003004	0.000026	0.045
650	9.035	0.014	0.002068	0.000008	0.023

Additional angle dependent integrating sphere discussion:

In Fig. 4c, the Fresnel equation for a material with refractive index n = 1.09 does not include the imaginary component of the refractive index (k) for two reasons. First, angle-dependent measurements were performed on 1 % nigrosine PDMS, showing that the total reflectance as a function of θ_i is consistent with the Fresnel equation for n = 1.4, which is the refractive index of pure PDMS (Supplementary Fig. 14b). This suggests that the inclusion of 1 % nigrosine did not significantly alter the refractive index of PDMS. Thus, assuming the microcavity optical response is derived from the bulk optical response of 1% nigrosine PDMS, the inclusion of k is not appropriate. Second, we attempted to include k in the Fresnel

equation under the assumption that k ≥ 0 and that the reflectance at θ_i = 0 ° aligns with measurements. However, we observed that at higher θ_i, equations with k increase more rapidly compared to those with n = 1.09 and k = 0 (Supplementary Fig. 14c).

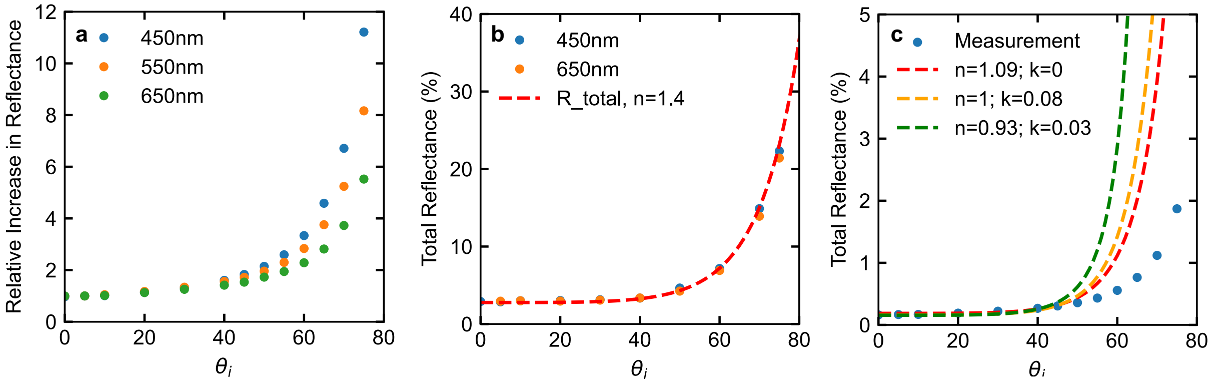

Supplementary Fig. 14. Angle dependent integrating sphere measurement on H15G1 PDMS and flat PDMS: (a) Relative increase in reflectance as a function of θ_i. (b) Total reflectance of 1% nigrosine PDMS as a function of θ_i and Fresnel equation with n = 1.4 (pure PDMS refractive index) under unpolarized illumination. (c) Additional Fresnel equation plot including the imaginary component of the refractive index (k).

Additional H15G1 sample BRDF presentation:

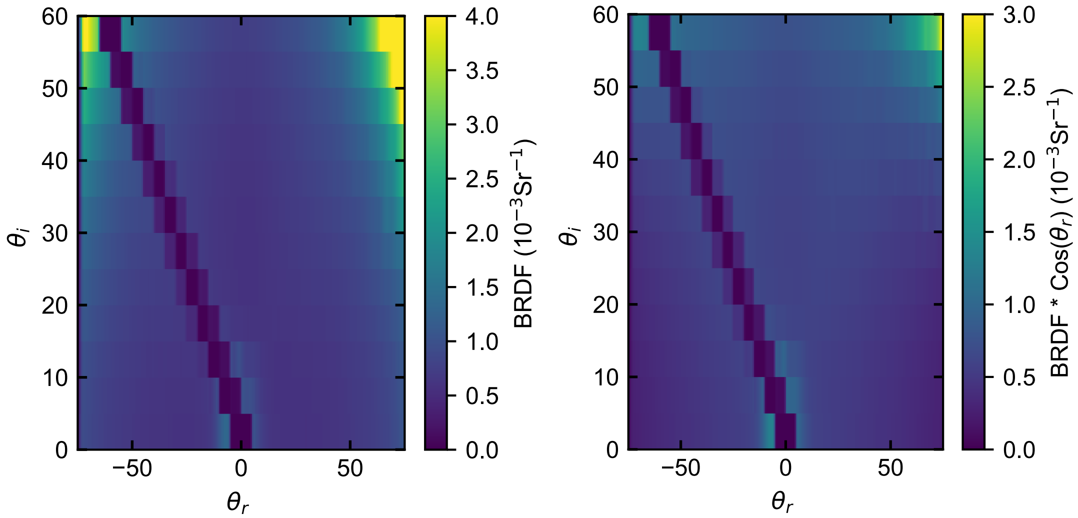

Supplementary Fig. 15. H15G1 BRDF measurement plotted using color scale of BRDF and BRDF * Cos(θ_r).

Black photo panel under varying illuminance:

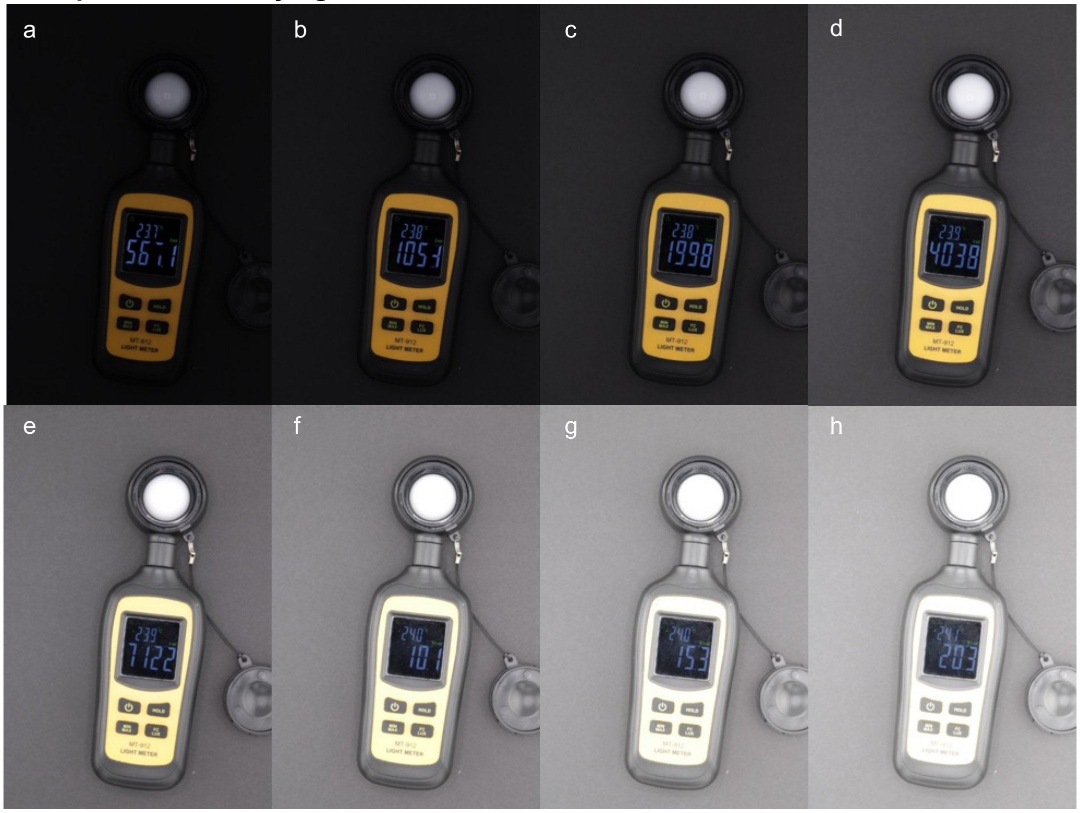

Supplementary Fig. 16. Black photo panel as a function of illuminance measured using a lux meter at consistent camera setting: (a) 567 lux, (b) 1054 lux, (c) 1998 lux, (d) 4038 lux, (e) 7122 lux, (f) 10100 lux, (g) 15300 lux, and (h) 20300 lux. The camera shutter speed is at 1/25 s and f number = f/5.6.

H15G1 robustness testing

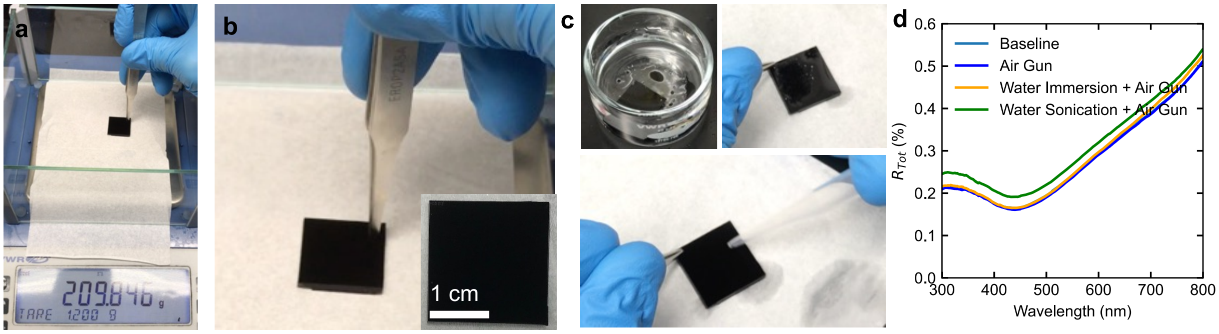

Supplementary Fig. 17. Indentation and immersion (sonication) test: (a) touching test by applying a ~2 Newton of pure normal load through a round head tweezer with a contact area of approximately 3mm^2 resulting a 600kPa. (b) zoomed in image of the touch test and photography of after touch test. (c) water immersion test and dry air blow test. (d) Integrating sphere measurement of the sample after damaging test.

Microcavity failure modes under shear loading:

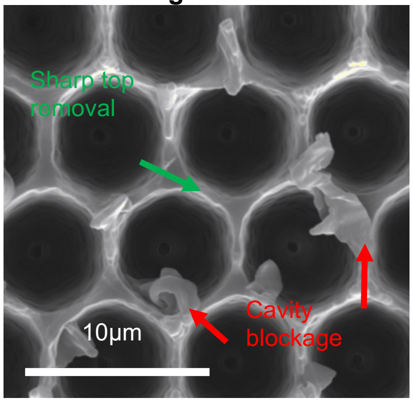

Supplementary Fig. 18. SEM image after a shear load is applied across the sample surface.

Photography of Robust H15G1 after damage:

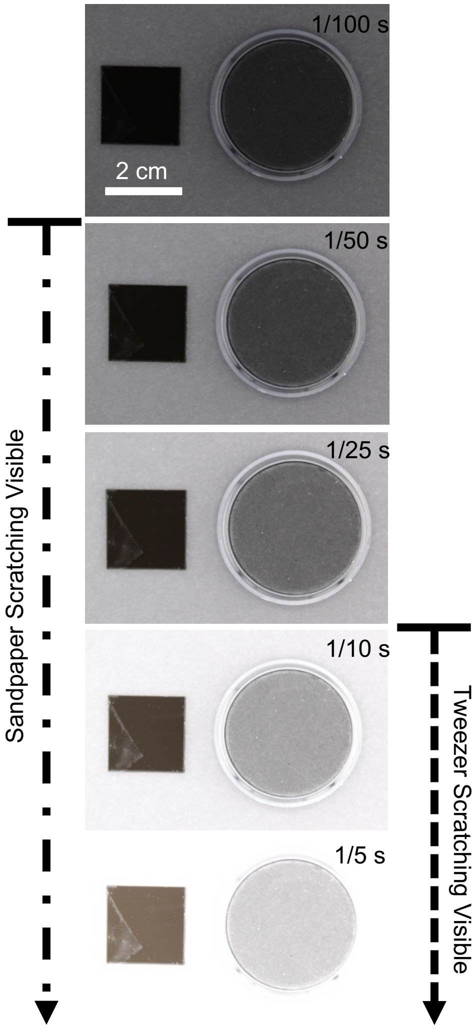

Supplementary Fig. 19. Photography image of the damaged robust H15G1 under 20,000 Lux illumination with varying shutter speed.

Abrasive material insertion:

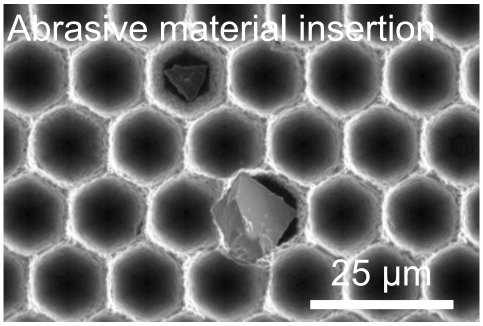

Supplementary Fig. 20. SEM of microcavity H15G1 with abrasive material insertion.

Robust H15 with varying gap total hemispherical reflectance measurements:

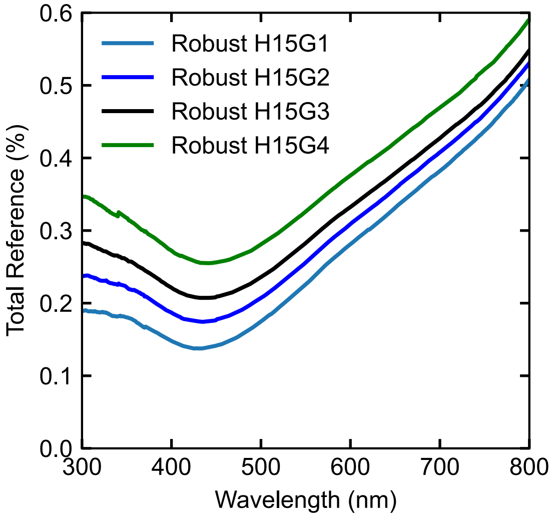

Supplementary Fig. 21. Total hemispherical reflectance measurement of robust H15 samples with varying gap size.

Supplementary Discussion 1: About Manufacturing
From a product manufacturing perspective, we wish to address key factors such as wafer size scalability, mold production costs, mold throughput, production line ownership costs, supply chain logistics, and ease of integration. These elements are critical for translating basic scientific research into commercially viable products. Without considering these factors, the technology is likely to remain confined to laboratory demonstrations rather than reaching full commercial applicability.

1. Silicon fabrication-based processes offer the potential to scale mold production from 100 mm to ≥200 mm diameter wafers, as 200 mm and 300 mm wafer production lines are common in industry-scale manufacturing. Currently, the limitation in wafer size is due to the constraints of the tools used for basic science research. In this work, we successfully scaled from a 75 mm diameter pattern to a 100 mm diameter mold with minimal investment in research and process development. Additionally, we are collaborating with external contractors to scale production from 100 mm to 150 mm diameter molds. While further process development will be required to scale production from 100 mm to 150 mm wafers, no additional investments in applied research are needed.

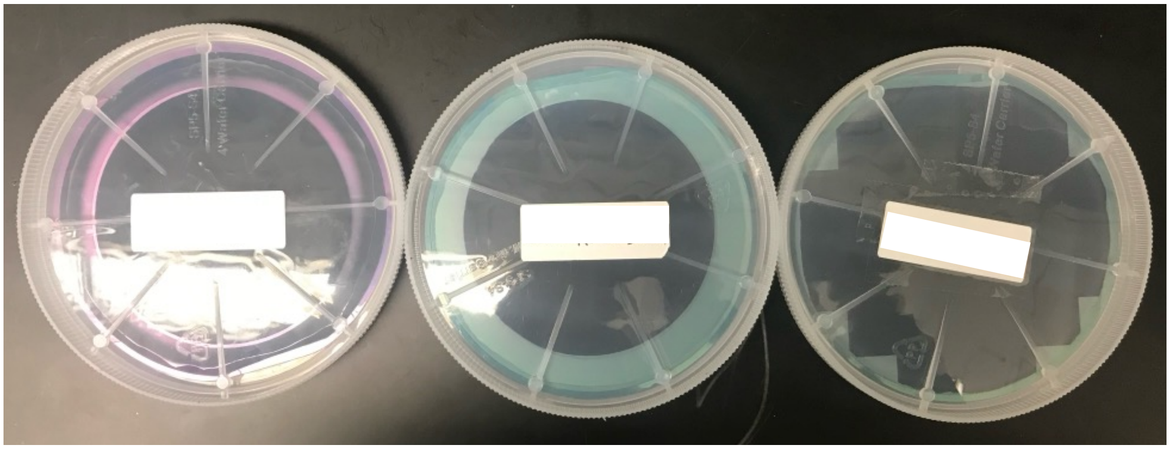

Supplementary Fig. 22. Demonstrating of scaling from 75 mm to 100 mm.

2. While the authors in Ref. 1 suggest that master mold replication could reduce costs, they did not demonstrate the feasibility of repetitive replication. In contrast, we include a cost analysis based on current labor market rates and process cost estimations (Supplementary Table 4). The conservative cost for fabricating an original silicon mold is estimated at under $500, with a mold throughput of less than 50 minutes per mold. This throughput can potentially be further improved by optimizing the DRIE etching parameters.

Supplementary Table 4. Fabrication cost and through put analysis.

	Time (minutes)	Cost per wafer ($)	Details
Skilled Labor	99.2	79.5	
Cleanroom Access	99.2	165.3	
Photoresist deposition	1	1.59	2mL
Lithography Tool	0.6	1.71	0.6 minutes
DRIE	45	85.6	45 minutes
Oxidation	3	0.04	300 minutes per 100 wafers
Bare Silicon Wafer	0	13.46	100 mm wafer
	Total Cost ($)	347.2	
	Throughput per Wafer (minutes)	49.6	Limited by number of DRIE

Supplementary Table 5. Basis of the coast analysis.

	Cost ($)	Unit	Cost	Unit	throughput	additional details
Skilled Labor	48.1	per hour	$100,000	per year		
Cleanroom Access	100	per hour				Cleanroom Access Includes basic chemicals
Photoresist	0.79	per mL	$3,000	per Gallon		
Lithography Tool	2.85	per minutes	$1,500,000	per Tool	100 wafers per hour through put	Referbished i-line stepper, Cost per minutes is based 1 year of use
DRIE Tool	1.90	per minutes	$1,000,000	per Tool		New, process upto 8" wafers, Cost per minutes is based 1 year of use
Wet Oxidation Furnace	228.3	per run	$500,000	per tube	100 wafers per run	New, each run take 5 hours including ramping and cooling, Cost per minutes is based 1 year of use
Bare Silicon Wafer	13.46	per wafer				

3. The use of an ion accelerator introduces drawbacks in terms of supply chain logistics, production line ownership costs, and ease of integration. Outsourcing the master mold production to a limited number of research facilities worldwide presents substantial risks for supply chain reliability. To have full control over the production process, one would need to own an ion accelerator, which can be prohibitively expensive. In contrast, the chemicals and tools required for silicon mold fabrication are widely available at universities, national labs, and through external contractors, facilitating easier integration into existing production facilities. Silicon fabrication methods enable production line owners to directly convert bare silicon wafers into molds for producing superblack

PDMS, reducing reliance on external facilities and providing greater control over the manufacturing process.
