# Supplementary material for: Engineering flexible superblack materials
Source: Nat Commun. 2025 May 19;16:4650. doi: 10.1038/s41467-025-59876-y (PMC12089317; doi:10.1038/s41467-025-59876-y)
Supplement: Supplementary file 2 — Description of Additional Supplementary Files [file 41467_2025_59876_MOESM2_ESM.pdf]

Description of Additional Supplementary Files

File name: Supplementary Movie 1
Description: Finger touch resistance of the R-H15G1.

File name: Supplementary Movie 2
Description: Scotch roller duster resistance of the R-H15G1.

File name: Supplementary Movie 3
Description: Tweezer scratch resistance of the R-H15G1.

File name: Supplementary Movie 4
Description: Surface hydrophobicity of the R-H15G1.
